# Supplementary material for: Older Adults’ Perspectives of Smart Technologies to Support Aging at Home: Insights from Five World Café Forums
Source: Int J Environ Res Public Health. 2022 Jun 25;19(13):7817. doi: 10.3390/ijerph19137817 (PMC9266000; doi:10.3390/ijerph19137817)
Supplement: Supplementary file 1 [file ijerph-19-07817-s001.zip › ijerph-1764876-supplementary.pdf]

Supplementary File for “Older adults' perspectives of smart technologies to support aging at home: Insights from five World Café forums”

Videos used as illustrative examples

Videos were used in the World Cafes to introduce participants to smart technologies which might be used to support older people living in the community

Autonomous vehicles

How driverless cars from CBC News, Canada, January 7th 2016, First 3.35 minutes

https://www.youtube.com/watch?v=XEebyt6G5kM

Robots

Eldercare robots, Voice of America, Sept 19 2018, First 2.54 minutes.

https://www.youtube.com/watch?v=P_0jzsNF8kA

Robot chores, Getty images TV, Dec 21 2017, First 1.48 minutes.

https://www.youtube.com/watch?v=3xWOzklsv-c,

I tried cleaning my entire apartment with robots, Home Insider, First 2 minutes.

https://www.youtube.com/watch?v=_20v6eMRQVg

Amazon Echo Alexa for people with disabilities, Assistive technology blog, First 3 minutes.

https://youtu.be/58eAWc1fRoQ

Smart Wearables/Homes

Consumer IOT, Elderly care. Genny.io, 2017. Complete video.

https://www.youtube.com/watch?app=desktop&v=BCK-v0cWE3k
